# Applications of the ROS-Responsive Thioketal Linker for the Production of Smart Nanomedicines

**DOI:** 10.3390/polym14040687

**Published:** 2022-02-11

**Authors:** Arianna Rinaldi, Riccardo Caraffi, Maria Vittoria Grazioli, Natalia Oddone, Luciana Giardino, Giovanni Tosi, Maria Angela Vandelli, Laura Calzà, Barbara Ruozi, Jason Thomas Duskey

**Affiliations:** 1 Nanotech Lab, Te.Far.T.I., Department Life Sciences, University of Modena and Reggio Emilia, 41125 Modena, Italy; arianna.rinaldi@unimore.it (A.R.); riccardo.caraffi@unimore.it (R.C.); mariavittoria.grazioli@unimore.it (M.V.G.); or noddone@pasteur.edu.uy (N.O.); gtosi@unimore.it (G.T.); mariaangela.vandelli@unimore.it (M.A.V.); barbara.ruozi@unimore.it (B.R.); 2 Clinical and Experimental Medicine PhD Program, University of Modena and Reggio Emilia, 41125 Modena, Italy; 3 Redox Biology of Trypanosomes, Institut Pasteur de Montevideo, Mataojo 2020, Montevideo 11400, Uruguay; 4 Interdepartmental Center for Industrial Research in Life Sciences and Technologies, University of Bologna, 40064 Bologna, Italy; luciana.giardino@unibo.it (L.G.); laura.calza@unibo.it (L.C.); 5 Department of Veterinary Medical Science, University of Bologna, 40064 Bologna, Italy; 6 Department of Pharmacy and BioTechnology, University of Bologna, 40127 Bologna, Italy

**Keywords:** thioketal, smart drug delivery systems, ROS-responsive biomaterials, nanomedicine, nanoparticles

## Abstract

Reactive oxygen species (ROS)-sensitive drug delivery systems (DDS) specifically responding to altered levels of ROS in the pathological microenvironment have emerged as an effective means to enhance the pharmaceutical efficacy of conventional nanomedicines, while simultaneously reducing side effects. In particular, the use of the biocompatible, biodegradable, and non-toxic ROS-responsive thioketal (TK) functional group in the design of smart DDS has grown exponentially in recent years. In the design of TK-based DDS, different technological uses of TK have been proposed to overcome the major limitations of conventional DDS counterparts including uncontrolled drug release and off-target effects. This review will focus on the different technological uses of TK-based biomaterials in smart nanomedicines by using it as a linker to connect a drug on the surface of nanoparticles, form prodrugs, as a core component of the DDS to directly control its structure, to control the opening of drug-releasing gates or to change the conformation of the nano-systems. A comprehensive view of the various uses of TK may allow researchers to exploit this reactive linker more consciously while designing nanomedicines to be more effective with improved disease-targeting ability, providing novel therapeutic opportunities in the treatment of many diseases.

## 1. Introduction

Nanoparticles (NPs) and nano-based drug delivery systems (DDS) (such as liposomes, polymeric nanoparticles, dendrimers, etc.) have the remarkable ability to minimize the degradation of therapeutic molecules, prevent harmful side-effects, as well as to increase the fraction of a drug that accumulates at the diseased site [1,2,3,4,5,6,7]; however, success of DDS as approved therapeutic treatments is still limited by certain shortcomings including: (1) instability during storage, leading to degradation or rapid escape of the drug; (2) non-specific bio-distribution; and (3) off-target effects or toxicity caused by uncontrolled drug release in circulation. These drawbacks lead to poor therapeutic effectiveness [8,9,10].

A promising approach to solve these flaws lies in the concept of creating smart-DDS. This can be done through various chemical/technological strategies aimed at developing core components that are stimuli-responsive. In this way, the smart DDS can be tuned dependent on some physio-pathological characteristic at the diseased site (e.g., pH, redox, temperature, mechanical force, ultrasound, etc.) to modulate the stability and drug release to increase therapeutic effectiveness while minimizing off-target effects [11,12,13,14,15,16].

Of these biological stimuli, reactive oxygen species (ROS, e.g., superoxide anion, hydrogen peroxide, and 2-hydroxyl radicals) have begun to gain attention. While playing a key role in regulating several physiological processes [17], their excessive intracellular accumulation has been shown to be the basis of numerous diseased states such as inflammatory diseases, neurodegenerative diseases, and cancer [18,19,20,21]. This difference in ROS levels between healthy cells and the pathological microenvironment has fueled the use of biomaterials (e.g., selenide/telluride-containing polymers, diselenide/ditelluride-containing polymers, polyoxalate, aminoacrylates, poly(proline), phenylboronic esters/acids, thioethers and thioketal) that are degraded or activated in specific environments [22,23,24].

Of particular interest, the thioketal (TK) moiety is being increasingly cited in the literature for new and innovative applications, especially in cancer and inflammatory diseases. TK is a subclass of the thioether group, which is cleaved to a thiol-containing group and acetone [25] upon exposure to ROS, while being considered biodegradable, non-toxic and amenable to incorporation into other polymers or conjugates [26,27]. The reactivity of TK to different types of ROS, as well as its biological reactivity, are well covered in the literature [22,25,28] and its prominence as a reactive linker has continued to increase. In this context, a growing number of studies suggest the advantages of using TK-DDS compared to conventional nanomedicine constructs [9,29].

In this review, we will introduce the chemistry of thioketals and their use as a reactive linker, but the main focus will be on the different technological uses of TK in the design of smart nanosystems that respond to ROS-rich microenvironments. This includes the incorporation of TK-based biomaterials not only into the structure of polymers that form the DDS core materials, but also as a ROS-reactive linker (to conjugate a drug to the surface of the DDS, or to develop TK-containing prodrugs where the drug is released upon ROS-induced cleavage of the TK). Moreover, the TK group has been used to control the opening of ROS-responsive gates in the structure of NPs, or to change the conformation of nanosystems after exposure to ROS. Finally, the ROS-responsiveness of TK-based biomaterials has been used in combination with other environmental stimuli to obtain dual or multistimuli-responsive DDS. An overview of the different ways to exploit TK in the development of smart DDS will be beneficial to select the proper use of this reactive linker to obtain nanomedicines with improved disease-targeting and minimized off-target effects.

## 2. Thioketal

**Figure 1:**
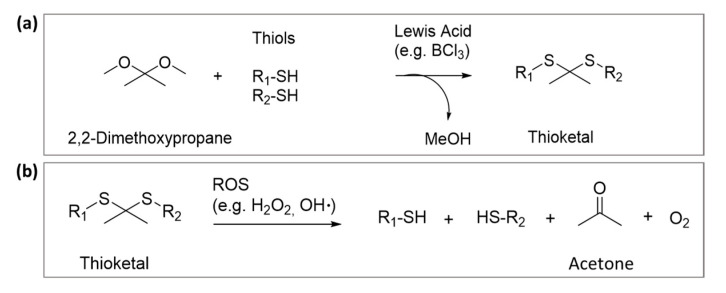
(**a**) Mechanism of synthesis of TK by condensation of thiols with ketones (readapted from numerous similar reactions from articles by El-Mohtadi et al. [26], McEnery et al. [30] and Ling et al. [31] and (**b**) chemical degradation of TK groups by ROS [25].

The TK functional group is a sulfur analogue of the ketal, whose synthesis can generally be carried out by the condensation of thiols with ketones (Figure 1a). Numerous examples exist of using this reaction in the literature where dimethoxypropane was condensed with 1,4-bis(mercaptomethyl)benzene [26], thioglycolic acid [30], or mercaptopropionic acid [31].

Advantages of the TK moiety include its stability to enzymes and acidic or basic environments; however it readily cleaved into its non-toxic thiol and acetone products when exposed to pathological levels of ROS in vitro or in vivo (Figure 1b) [25]. The susceptibility of TK to various ROS, the degradation kinetics and other physico-chemical characteristics, including stability to acidic or basic pH, have been extensively reviewed in the literature and will not be the focus of this review [27,32,33].

Due to their potential use as disease-selective moieties, TK-containing materials have been studied for different applications including tissue engineering and regenerative medicine, for example, biodegradable scaffolds designed to be specifically degraded by ROS in the diseased site [30,34,35]; however, one of the most cutting-edge applications of TK lies in the development of ROS-responsive DDS with enhanced pharmaceutical efficacy compared to conventional nanomedicine counterparts. 

## 3. Different Technological Uses of TK in Drug Delivery Systems

To take advantage of ROS-specific cleavage, TK groups have been integrated into polymer chains or used to create responsive linkers for the development of selective and improved “smart” nanosystems. This offers the advantage of controlling the degradation kinetics, drug release kinetics, or even increasing potential administration routes (e.g., TK stability to pH and enzymes has been used to improve systemic administration) [33,36].

To better understand how TK groups can improve the therapeutic effectiveness of these smart DDS, it is important to have an overall view of its various uses in ROS reactive nano-based systems. Here, we will provide an overview of DDS where TK moieties have been: (1) used as linkers in DDS conjugation, (2) incorporated into polymer structures or (3) exploited to obtain ROS-responsive gates or change the surface characteristics of the DDS. 

### 3.1. TK Used as a Linker in DDS Conjugation for On-Demand Drug Release

One possible use of TK in designing ROS-specific DDS consists of covalently attaching it to drug, dye, or biologically active molecules to obtain a linker that causes its release upon ROS-specific cleavage. In this way, therapeutic efficacy can be improved with minimized off-target effects by releasing the conjugated drug in a controlled manner [37].

#### 3.1.1. TK as Linker to Attach Drugs to the Surface of Nanoparticles

**Figure 2:**
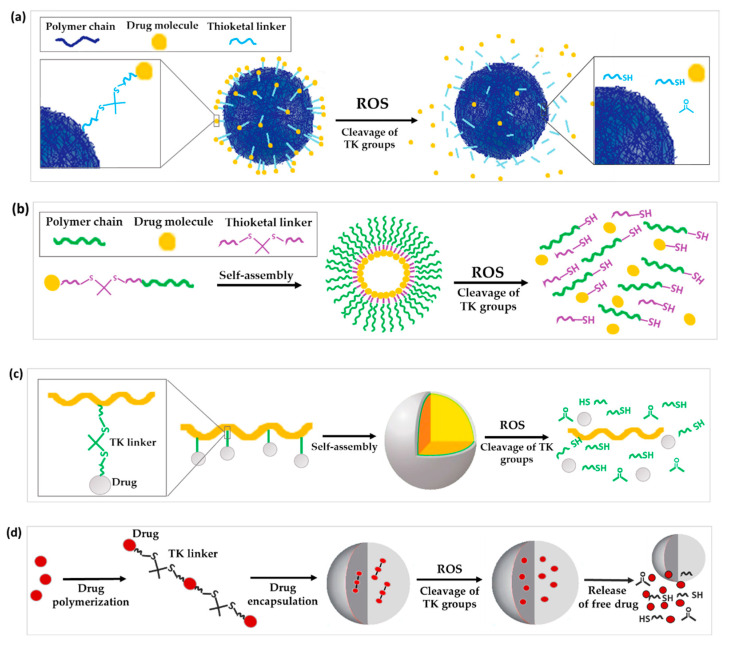
Schematic illustration of the chemical structure and corresponding release mechanisms of TK-based ROS-responsive nanosystems, where TK is used as (**a**) linker to attach drugs to the surface of nanoparticles, (**b**) linkage to form prodrug-based NPs, (**c**) linker to form polyprodrug-based NPs with drug molecules attached to the same polymer unit by TK linkages or (**d**) linker to form encapsulated polyprodrugs based on multiple drug molecules attached to each other via TK bridges.

As a first approach, TK linkers have been exploited for the covalent attachment of drugs to the surface of inorganic NPs (Figure 2a). For instance, Shi et al. developed a novel surface-functionalized fullerene (C_60_)-based DDS targeted to tumors with the Asn-Gly-Arg peptide designed to with an “off-on” state to maximize the antitumor efficacy of the nanosystem. In this work, doxorubicin was attached to C_60_ via a ROS-sensitive thioketal linker. When in its “off”-state the doxorubicin remained firmly bound to the surface while light activation (the “on”-state) led to ROS production, the breakdown of the TK linker and subsequent drug release [38].

#### 3.1.2. TK as a Linker for Polymer-Dye Conjugation

Alternatively, TK has been used as a linker to form polymer-dye conjugates. Recently, the synthesis of a fluorescent conjugate between Cy5, a near-infrared fluorescence-emitting dye, the TK moiety, and polyethylene glycol monomethyl ether (mPEG) (mPEG-TK-Cy5) was reported. The aim was to perform proof-of-concept studies that confirm the selective release of this dye in cancer cells respective of normal cells. In ROS solutions it was observed that the presence of TK blocked Cy5 quenching. Further in vitro studies using rat glioblastoma cells (C6) with high intrinsic levels of ROS demonstrated the release of Cy5, while no release was achieved in normal astrocyte cells with physiological levels of ROS (DI TNC1) [39].

#### 3.1.3. TK as Linkage to Form Prodrug or Polyprodrugs

Prodrugs are inactive precursors of a drug designed to increase the amount of drug converted to its active form by a certain pathological or biological stimulus [40,41,42]. With the prodrug idea in mind, ROS-sensitive TK linkers have been exploited to conjugate a drug to another drug or polymer that can then self-assemble into NPs [43]. Once the prodrug NPs reach the diseased tissues, the high concentration of ROS cleaves the TK linker to release and activate the drug (Figure 2b). 

In order to treat inflammatory bowel disease, Li et al. used an aromatized TK to conjugate budesonide (an anti-inflammatory drug [44]) with the antioxidant Tempol to improve colon delivery. Interestingly, π-π stacking interactions of the TK linker, as well as hydrophobic interactions between budesonide moieties promoted self-assembly of the prodrug into NPs. In fact, authors were able to calculate the critical aggregation concentration of the prodrug as 16 µg/mL, which is low thanks to the strong non-covalent hydrophobic interactions. Ultimately, hydroxide-triggered cleavage of TK activated the drug and achieving anti-inflammatory, antioxidant, and anti-apoptotic effects in an inflammatory bowel disease mouse model [45]. Other examples regarding TK-containing prodrugs included improving the solubility and pharmacokinetic half-life of chemotherapeutic drugs by conjugating them to polyethylene glycol (PEG) [46]. For instance, melphalan (MPH) was conjugated to PEG via TK to form a prodrug that self-assembled into micelles for treating glioblastoma multiforme. The incorporation of the TK linker enabled selective ROS-triggered release of MPH in cancer cells increasing the anticancer activity and cytotoxicity in rat C6 and human U251 MG glioblastoma cells compared to the non-ROS sensitive prodrug mPEG-MPH. On the other hand, TK was not cleaved and did not induce cytotoxicity in healthy DI TNC1 cells [43]. In another study, Pan et al. synthesized a PEG-doxorubicin (DOX) conjugate via a TK moiety that self-assembled into prodrug NPs that showed negligible release of DOX in HepG2 cells but significantly enhanced drug release (45.5% in 24 h) in the presence of 100 μM H_2_O_2_, indicating the tumor-ROS-responsiveness of NPs selectively in ROS-rich environment. Furthermore, these nanosystems showed higher antitumor activity than free DOX in liver cancer (HepG2)-bearing nude mice [47]. In addition, Lin et al. synthesized a PEG-TK-mitoxantrone prodrug that co-assembled with lipid-PEG to form NPs embedding a cisplatin prodrug in the core for combined cancer therapy. Release studies showed that about 80% of cisplatin prodrug and 40% of the loaded mitoxantrone (MTO) were rapidly released from NPs within 24 h when incubated in PBS solution containing 100 μM KO_2_, which is a biologically relevant intracellular ROS concentration. Moreover, when the TK group was replaced with a non-responsive carbon–carbon bond no drug release was observed from the NPs at 100 μM KO_2_, and no difference was observed in their release profile compared to NPs incubated without ROS. In vivo, the environmental responsive cleavage of the TK led to localized release of both the MTO and cisplatin creating a synergistic tumor growth inhibition in a prostate cancer mice model [48]. 

TK linkers have also been applied for the development of polyprodrugs where: (a) drug molecules are attached to the same polymer unit by TK linkages or (b) drug molecules are attached to each other by means of TK bridges (Figure 2c,d) [49,50]. Regarding the first type of polyprodrug, Yin and co-workers conjugated camptothecin (CPT) molecules to co-block polymers from PEG and polymerized methacrylate monomers by linking the drug to the side chains of methacrylate units through a TK linker. These co-block polymers self-assembled into micelles encapsulating β-lapachone (Lapa), named Lapa@NPs, for cancer application [51]. Regarding the second type of polyprodrug, Xu and co-workers polymerized multiple molecules of MTO by TK linkers. This polyprodrug self-assembled into NPs which were further surface decorated with the RGD (arginine-glycine-aspartic acid) peptide to impart tumor-targeting ability. These NP- prodrugs showed high and stable drug loading. The polymerization of MTO prevented the escape of drug molecules from NPs, and the absence of ROS led to negligible drug release in vitro. In contrast, 25% and 40% of loaded MTO was released from NPs when incubated in PBS buffer containing 50 μM and 100 μM of KO_2_ solution for 48 h, respectively. TK bonds in the structure of the polyprodrug ensured a ROS-dependent chain-breakage and the consequent release of active MTO in cancer cells [52].

### 3.2. TK Incorporated into the Polymer Structure

A major goal of nanomedicine is to develop stable nanocarriers that protect a drug during its passage through the bloodstream and release the payload in the diseased site via controlled degradation of the DDS. Although such a design concept is well known, most of the conventional nanocarriers do not adequately fulfill these requirements, resulting in premature and/or off-target drug release [53]. TK’s specificity for being degraded by ROS, and the higher ROS concentrations in several diseased states, makes it a prime candidate. In this regard, the incorporation of a ROS-sensitive TK moiety into the polymer backbone of the nanosystem has emerged as a promising strategy to control degradation and release kinetics, and to release the payload specifically in the diseased area. In the reported studies, controlled degradation and drug release normally occur by ROS-triggered cleavage of the TK chemical bonds leading to polymer backbone decomposition.

**Figure 3:**
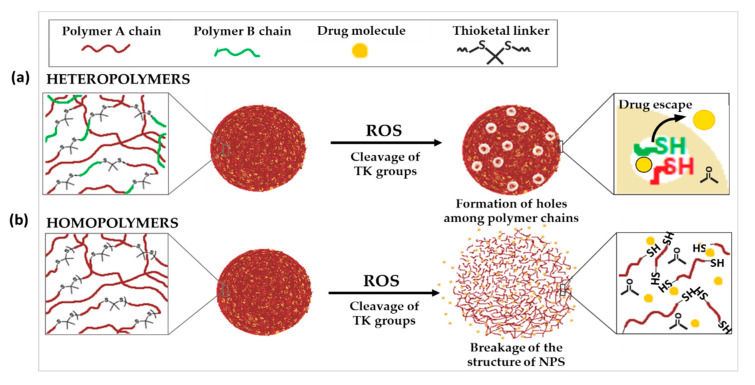
Schematic illustration of the chemical structure and corresponding release mechanisms of TK-based ROS-responsive nanosystems, where TK is incorporated into (**a**) heteropolymers, two polymers with different physico-chemical characteristics or (**b**) homopolymers.

To fulfill these objectives, some works reported the incorporation of TK linkages into homopolymers. For instance, Kim et al. synthesized poly(1,4-phenyleneacetone dimethylene thioketal) polymer (PPADT). PPADT was used to prepare polymeric NPs loaded with Nile red as a model drug. The molecular weight of PPADT dramatically decreased to 28.8% over 24 h after incubation in a 10 mM KO_2_ solution. This is compared to 92% of the polymer remaining intact over 96 h in the absence of the TK linker. While the drug released from the NPs was negligible in the absence of ROS after 60 h, it reached 73% in presence of TK and 10 mM ROS. Furthermore, no significant change in the hydrodynamic diameter of the NPs was observed during exposure to ROS. This suggests that the degradation release of the drug was not caused by the destruction of the core, but rather by holes within the matrix caused by the destruction of the polymer backbone bonds (Figure 3a) [33]. Similar results were observed in two other studies by Wang et al. and Wilson and co-workers. PPADT was exploited to develop NPs loaded with stromal cell-derived factor 1α (SDF-1α), a chemokine that plays a key role in wound treatment, or a small oligonucleotide (siRNA) that inhibits the expression of the proinflammatory cytokine tumor necrosis factor-α (TNFα) [36,54]. All three of these drug-loaded NPs displayed significantly higher therapeutic effectiveness in vivo compared to nanoparticles without TK. 

As an alternative to the incorporation of TK into homopolymers, it has also been used to link together two polymers with different physico-chemical characteristics. In contrary to the previous cases, where the ROS-triggered cleavage of TK groups did not cause a significant change in the size of NPs, other examples demonstrated huge increase in nanoparticle diameter after exposure to ROS. This could suggest that the cleavage of TK resulted in the complete breakdown of the NP structure (Figure 3b).

Two examples can be found in the studies carried out by Li et al. In the first study, they conjugated poly (lactic-co-glycolic acid) (PLGA) with methoxyl-polyethylene glycol (MeO-PEG) by TK linker to form a diblock co-polymer that self-assembled into NPs loaded with DOX. In the second study, MeO-PEG was replaced by PEG and DOX was co-loaded into NPs with α-tocopheryl succinate, which stimulates ROS production into cells. In both cases, the 6 h and 48 h cumulative release of DOX from NPs was significantly higher when NPs were incubated with 50 μM ROS, which increased significantly to more than 80% after 48 h when incubated in 100 μM. This increase in DOX release rate was attributed to the cleavage of TK linker resulting in the breakdown of the NPs increasing their size and releasing DOX. In contrast, no morphological changes were detected in the absence of ROS or in control drug-loaded NPs without the TK linker. Moreover, co-loading alpha-tocopheryl succinate with DOX led to enhanced antitumor efficiency, mainly due to the higher intracellular ROS levels in cancer cells induced by alpha-tocopherol which promoted the cleavage of TK [55,56]. In another work, Sun et al. used a TK linker with a π-conjugated structure between methoxy polyethylene glycol (PEG) and poly(ε-caprolactone) (PCL) chains. mPEG-TK-PCL self-assembled into micelles which were loaded with doxorubicin. π–π stacking and hydrophobic interactions between TK moieties and DOX enhanced the drug loading content of micelles (12.8%) with respect to mPEG-PCL micelles without TK linker (8.6%). While the size of DOX-loaded micelles remained unchanged in PBS, it increased to more than 1000 nm when the micelles were put in ROS-rich microenvironment for 48 h. This increase in size could be attributed to the ROS-responsive degradation of the polymer leading to breakdown and swelling of the core structure with subsequent release of DOX. This selective ROS-responsive drug release helped lower in vivo toxicity towards normal cells and remarkably enhanced antitumor efficacy of DOX-loaded micelles [57].

### 3.3. Other Technological Uses of TK in DDS Structure

Along with releasing a conjugated pharmaco or destabilizing the DDS structure to control release, other interesting technological uses of TK have emerged. Among them, TK-based biomaterials have been used to control the opening of drug-release gates, or to change the surface characteristics or the conformation of NPs upon ROS exposure.

#### 3.3.1. TK-Based ROS-Responsive Gates to Prevent Premature Drug Release

**Figure 4:**
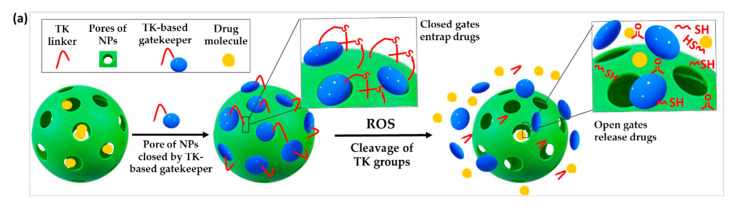

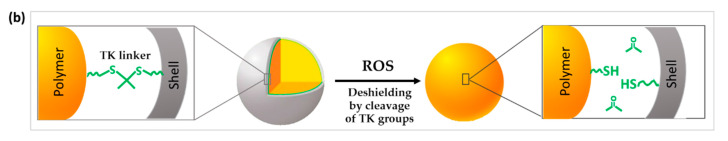
Schematic illustration of the chemical structure and corresponding release mechanisms of TK-based ROS-responsive nanosystems where TK is used to (**a**) create ROS-responsive drug-releasing gates or (**b**) induce the surface deshielding in response to ROS.

Stimuli-responsive gatekeepers are structures able to keep the pores of NPs closed under physiological conditions and open them on-demand after the application of a particular stimulus, in this case the upregulated ROS levels in the pathological site [58,59]. The use of ROS-responsive gates based on TK has been proposed to prevent the premature release of drugs from NPs (Figure 4a).

For instance, Shi et al. developed a novel type of mesoporous titanium dioxide NPs. After loading the anticancer drug docetaxel in the pores of the NPs, they were blocked by attaching β-cyclodextrin (β-CD) to the outer surface via a TK linker. The obtained NPs selectively released the drug only after ultrasound-induced ROS production which cleaved the ROS-sensitive linker and detached the gatekeepers [60]. Similar experiments were performed by Hu et al. using mesoporous silica nanoparticles (MSNs) for the co-encapsulation of DOX and the ROS producing agent α-tocopheryl succinate (α-TOS). These drugs were similarly blocked in the MSN pores by conjugating a TK-β-CD conjugate to the surface of the NPs. Less than 20% of DOX was leaked after 72 h of incubation in absence of ROS, proving the efficiency of the gating procedure. In contrast, 60% drug was released after a 72 h incubation with 100 μM H_2_O_2_. When dosed into human breast cancer (MCF-7) cells, increased intracellular ROS induced by the co-release of α-TOS further triggered the cleavage of the TK linker and release of DOX for enhanced chemotherapeutic effect [61].

#### 3.3.2. TK Used to Change the Surface Characteristics or the Conformation of the DDS 

As shown in the previous section, TK functional groups have been used to prepare DDS that degrade or release conjugated drugs from prodrugs or NPs upon ROS stimulation, however, TK moieties have also been exploited to design DDS that change surface characteristics or their conformation with the aim of improving cell-uptake and site-specific drug delivery in response to ROS. 

In this context, Li et al. developed a nanosystem that changes the surface characteristics by controlling polyethylene glycol (PEG) shielding/deshielding at the desired site of action (Figure 4b). Paclitaxel (PTX) and chlorin e6 (Ce6) were co-encapsulated into NPs formed by the self-assembly of a diblock copolymer of PEG conjugated with poly(D,L-lactic acid) (PLA) via a TK bridge (PEG-TK-PLA NPs). While PEGylation of PEG-TK-PLA NPs prolongs their circulation time in blood, the exposure to high levels of ROS induced the breakage of the TK groups and the rapid loss of the PEG corona shell. This was was demonstrated by measuring the amount of released thiol-terminated PEG residues released after TK cleavage by Ellman’s reagent assay. In contrast, a negligible deshielding was observed in control PEG-PLA NPs. PEG deshielding did not affect the size or the morphology of the NPs but significantly enhanced cellular uptake, probably due to a lower surface PEG density [62]. In this study, enhanced levels of ROS sufficient to cleave the TK groups and achieve deshielding were produced after Ce6 activation triggered by the exposure to 600-nm light irradiation. This provides an example of multi responsive DDS which will be discussed more in detail in Section 4.1.

Regarding the application of TK to change the conformation of NPs, an example is provided by Cheng et al. They modified the side chains of polyvinyl alcohol (PVA) with cytotoxic mitochondrial targeted KLAK peptides and diblock co-polymers of a second peptide (KLVFF) conjugated to PEG by means of TK linker (KLVFF-TK-PEG). The modified PVA self-assembled into NPs, intended to specifically target mitochondria in cancer cells, while once inside the diseased cells the hydrophilic PEG chains were cleaved due to high levels of intracellular ROS. PEG detachment induced a morphology switch of the NPs into fibrous structures which exposed multiple KLAK molecules on the surface. KLAK then interacted with the mitochondria and provoked their disruption in cancer cells, ensuring high cytotoxicity in in vitro and in vivo models [63].

## 4. TK-Based ROS-Responsive DDS in Association with Other Stimuli

**Figure 5:**
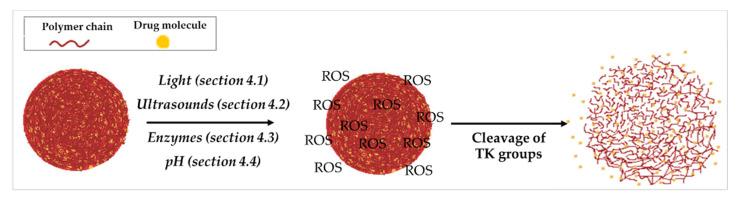
Schematic illustration of the release mechanism of a TK-based DDS in association with other stimuli.

To improve treatment efficacy and reduce side effects, TK-based DDS responsive to dual or multiple stimuli were developed to more precisely control cargo release profiles at the target site. While the most widely used TK co-stimulus is light, various other stimuli have been exploited, such as temperature, sonodynamic therapy, enzymes, or pH (Figure 5). 

### 4.1. Light

Photodynamic therapy (PDT) is a therapeutic treatment based on destroying diseased cells via ROS formation upon light irradiation of a photosensitizer (PS). The activation of a PS by specific wavelengths of light causes photochemical reactions that, in the presence of oxygen, result in the formation of ROS and consequent cell death [64,65].

**Figure 6:**
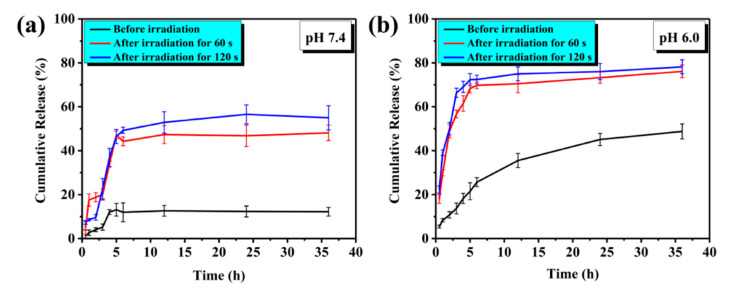
Effect of light on drug release from photosensitive TK-based DDS at different pH. Cumulative release profile at (**a**) pH 7.4 and (**b**) pH 6.0, comparing irradiated to non-irradiated DDS. Reproduced from the open source journal of International Journal of Molecular Sciences; published by MDPI; 2021 [69].

Synergistic combinations of chemotherapy and PDT have been exploited to enhance the therapeutic efficacy of TK-based smart nanomedicines [66,67]. To this aim, anti-cancer drugs and PS have been co-encapsulated into the same TK-based DDS. Under light irradiation, ROS produced by the PS not only can be used for PDT, but also can cleave the TK linker to more rapidly or completely release the chemotherapeutic agent. Being an exogenous stimulus, light does not depend on the variation of tumor microenvironment and can be easily controlled in terms of wavelength, intensity, exposure area, and treatment duration to achieve spatiotemporal drug release (Figure 6) [68,69]. This synergistic approach was found to be particularly advantageous in the application of TK-based nanomedicines in cancer. In fact, while tumor tissue ROS concentrations are elevated, this concentration is highly variable between cancer types and are often insufficient to completely cleave TK based DDS, resulting in a slow or non-complete drug release and consequently low effectiveness [70]. In addition, dual selectivity in therapy can be obtained by developing DDS that ensure PS accumulation and light irradiation selectively on the diseased tissues [65]. Several photosensitizers have been exploited for the formulation of dual ROS and light-responsive DDS, including Ce6 [65,71] and Meso-tetraphenylporphyrin (TPP) [72].

The advantages of dual ROS and light-responsive TK-based DDS have been described in numerous works. In these studies, TK has been incorporated into the nanocarriers by means of different techniques that incorporate all the previously described TK uses to enhance their responsiveness. 

#### 4.1.1. TK as Linker in DDS Conjugation and Light

ROS-sensitive TK linkers have been used for drug-to-polymer or drug-to-drug prodrugs that self-assemble into NPs for the co-encapsulation of an anticancer drug and a photosensitizer.

Regarding polymer-TK-drug prodrugs, Yue and co-workers prepared NPs loaded with the photosensitizer zinc phthalocyanine (ZnPc) from the self-assembly of the mitochondria-targeting triphenylphosphonium (TPP) to one side of polyethylene glycol (PEG) and CPT via a TK linker to the other side (CPT-TK-PEG-TPP). After accumulation of the NPs in cancer cell mitochondria, light-induced production of ROS by ZnPc not only exerted a cytotoxic effect, but also cleaved the TK linker provoking the release of CPT. While 34% of the drug was released in 4 h upon laser irradiation, showing anticancer efficacy in lung cancer animal models, NPs not subjected to laser irradiation showed no drug release during the same period [73]. In another study, Phua and co-workers exploited the strong binding of beta-cyclodextrin (β-CD) and adamantane (ADA) to form complexes via noncovalent bonds based on host-guest interactions. β-CD molecules formed inclusion complexes by interacting with (1) adamantane-modified CPT prodrug caged via a TK linker (CPT-TK-ADA), and (2) adamantane-modified photosensitizer (aPS) (PS-TK-ADA) via host-guest interactions. This supramolecular NP self-assembly encapsulated the prodrugs and, upon irradiation, the aPS produced ROS, which cleaved the TK linker between the adamantane and CPT, which resulted in a cascade release of free CPT. This controlled released led to significant tumor regression in in vivo tumor-bearing mice models after intravenous injection [74]. In another work, Pei et al. covalently conjugated DOX to the side groups of polyphosphoester via ROS-sensitive TK bonds. The polymer-TK-drug co-self-assembled with the photosensitizer Ce6, which produces ROS by activation upon light irradiation to enhance ROS levels in the diseased site. The TK bond was stable in physiological conditions and prevented premature drug leakage at off-target sites. On the other hand, ROS produced in cancer cells by activated Ce6 induced selective cleavage of the TK moiety, ensuring site-specific release and activation of free DOX and significantly enhancing the therapeutic efficacy with minimized side effects in vivo [53]. Finally, Shi et al. synthesized a PEGylated diacetoxyl-TK prodrug by linking DOX to a PEG and RGD conjugate. The obtained prodrug self-assembled into NPs co-encapsulating the photosensitizer hematoporphyrin (HP) for oral tongue squamous cell carcinoma treatment. DOX release from the NPs in vitro (CAL-27 tumor cells) was faster upon laser irradiation than when non-irradiated due to the ROS-induced cleavage of TK. In vitro, targeted and non-targeted NPs loaded with HP and DOX showed more potent cytotoxicity than free HP, suggesting that most of DOX was released through the cleavage of TK. On the other hand, RGD-mediated targeting was shown to enhance specific uptake of the NPs. In mice bearing CAL-27 tumors, DOX/HP-loaded NPs showed significantly enhanced inhibitory effects on tumor growth compared to both free DOX and free HP with laser irradiation, which exhibited strong synergistic effects between the PDT and chemotherapy [75].

#### 4.1.2. TK Incorporated into Polymers and PDT

The combination of PDT and the ROS-responsive behavior of TK has also been exploited when TK is incorporated into the polymer backbone of NPs. For instance, in a study carried out by Seah et al., photodynamic generation of ROS by meso-tetraphenylporphyrin was exploited to control the release of PTX from micelles formed by the self-assembly of a triblock copolymer containing TK groups. The generated ROS cleaved the TK groups leading to the formation of pores in the polymer core. Most of the encapsulated PTX was burst-released through the pores arriving at a maximum of ∼70% over 12 h. This is compared to only 17.5% of the drug being released over three days without PTD. Moreover, a decrease in the loading yield of meso-tetraphenylporphyrin in NPs accelerated the release of PTX, thus suggesting the possibility a way to modulate the release rate of encapsulated drugs. Loaded micelles showed a high efficiency in tumor growth suppression without any significant premature drug leakage [76,77]. In another study, Sun et al. explored a PEGylated polyphosphate polymer containing thioketal groups to form self-assembled NPs. Ce6 and DOX were simultaneously loaded into these NPs and when subjected to 660-nm laser irradiation. ROS produced by the activation of Ce6 not only induced tumor cell death, but also cleaved the TK linkers in situ resulting in the rapid degradation of NPs and accelerated DOX release. On the contrary, the control NPs that did not contain Ce6 or were loaded with DOX and Ce6 but without ROS-cleavable linker showed minimal degradation. This synergistic approach resulted in superior anticancer activity even in a DOX-resistant tumor model with nearly 60% of MCF-7/ADR cells being destroyed at 72 h at the highest DOX concentration [78].

Similarly, Li and co-workers co-encapsulated DOX and Ce6 into micelles from self-assembled polyethylene glycol (PEG)-diaxophosopholane (PBYP). To prevent the immature release of the payload from NPs, the polymer chains forming the core of NPs were cross-linked via TK linkages. As a result, the release of DOX from the micelles was quite slow in the absence of ROS or at 0.1 mM H_2_O_2_ (intracellular concentration in cancer cells), indicating that natural cellular ROS levels were insufficient to trigger the de-crosslinking of micelles and drug release. Under light irradiation, activated Ce6 enhanced ROS concentration in cancer cells and accelerated drug release in response to ROS via breakage of the TK cross-linker and subsequent micelle destabilization [79]. Alternatively, Jin et al. designed hyperbranched polyphosphoesters monomers (HBPTK) cross-linked with TK units end-capped with Ce6. The amphiphilic structure self-assembled into micelles loaded with the anticancer drug CPT. Upon laser irradiation, ROS generated by Ce6 triggered cleavage of the TK units, which not only facilitate faster diffusion of the NPs by reducing their size, but also induce the cascade release of CPT. Combining chemotherapy and photodynamic therapy, micelles exhibited the highest tumor inhibition rate (96%) in mice tumor models [80].

#### 4.1.3. TK-Based ROS-Responsive Gates and Light

Dual ROS and light-responsive DDS have also been developed by using TK to create ROS-responsive gates. In this regard, Zhang and co-workers used mesoporous silica coated NPs for the co-loading of ce6 and DOX, with subsequent gating of NPS achieved by TK linkages with silane groups. Without near-infrared radiation, TK gates largely hamper the release of DOX (below 15 % in 300 min). Drug release further decreased (8.0% in 300 min) by increasing the number of TK gates. However, upon light irradiation, the drug release almost tripled because of Ce6-induced production of ROS [67].

#### 4.1.4. TK Used to Change the Surface Characteristics of the DDS and Light

The synergistic combination of TK and PDT has been exploited to induce a change of the core characteristics of NPs by the deshielding process after exposure to increased levels of ROS generated by the light-activated photosensitizer.

For instance, Mohammed and co-workers used two di-block copolymers, one containing a TK linkage and a long poly(N,N-dimethylacrylamide) (PDMA) block (PCL-TK-PDMA), and a second one containing the small cell-penetrating block poly(2-guanidinoethyl methacrylate) (PGEMA), namely PCL-PGEMA. These di-blocks were used to prepare self-assembled micelles for the encapsulation of PTX and Ce6. Hydrophilic PDMA shells on the surface of the NPs shielded the cellular internalization of PGEMA to increase the half-life circulation in the blood. Upon light irradiation, light-induced production of ROS triggered TK cleavage leading to the PDMA block deshielding while exposing the PGEMA block on the surface of the micelles to enhance cell uptake. The cleavage of TK, studied by gel permeation chromatography analysis, achieved a ratio of 29% which gradually increased to more than 40% under continuous irradiation. In contrast, no degradation was observed in the absence of light irradiation. Deshielding was also confirmed by a size decrease and ζ-potential increase (from 1.8 to 13 mV) of the NPs under light irradiation which were presumably attributed to the partial removal of PDMA shells and exposure of positively charged PGEMA shells [81].

#### 4.1.5. TK-Containing Nanocomplexes for Gene Delivery and Light

PDT has also been combined with gene therapy, exploiting the presence of TK moiety in NPs to provide spatial and temporal control of gene delivery. For instance, Wang and co-workers developed a DDS for the delivery of a small interference RNA (siRNA) by conjugating it to branched polyethylenimine (PEI) via a TK linker to obtain TK-PEI. This was then decorated by the photosensitizer Ce6 via an amidation reaction (TK-PEI-Ce6). The obtained TK-PEI-Ce6 efficiently bound siRNA to form a nanocomplex. Light-triggered generation of ROS accelerated endosomal escape of the nanosystem within 6 min by oxidizing and destroying the endosomal membranes. ROS-induced disruption of TK linker provided light-triggered intracellular release and superior antitumor efficacy [82]. Similarly, TK-PEI polymer was condensed with plasmid DNA encoding p53 to form nanocomplexes. Hyaluronic acid (HA) was modified with the photosensitizer pheophytin a (Pha), and the obtained negatively charged HA-Pha was coated onto the NPs. A first short-time (8 min) light irradiation facilitates p53 gene release via degradation of TK-PEI. The same light irradiation at a longtime duration (30 min) generated lethal amounts of ROS to cooperatively kill cancer cells with p53, achieving high anti-cancer efficacy by dual-step light irradiation [83].

### 4.2. TK and Ultrasounds

As an alternative to light, ultrasounds have been proposed as a stimulus to combine with chemotherapy in dual stimulus-responsive DDS. Sonodynamic therapy (SDT) exploits ultrasound-induced production of ROS by activating a non-toxic sonosensitizer in the presence of molecular oxygen, following the same concept as photodynamic therapy [84]. Compared to light, ultrasounds can penetrate deeper into tissues and can be focused into a smaller region of a tumor, offering an alternative strategy to non-invasively eradicate solid tumors in a site-directed manner [85]. As in the case of photodynamic therapy, a synergistic combination of SDT and chemotherapy was used in TK-based smart nanomedicines to boost drug release or to enhance the cytotoxic effect of the chemotherapeutic drug selectively on the diseased cells. 

In recent work, Wu et al. synthesized a PEGylated prodrug of PTX, where PEG and the drug are linked by TK moiety, that self-assembled into NPs loading the sonosensitizer IR780 in the core (IR780/PTL-NPs). Under ultrasound stimulation, sonosensitizer activation in NPs produced high amounts of ROS, which boosted PTX release by breaking the TK groups. Drug release by dialysis from IR780/PTL-NPs was significantly higher (66.6% at 30 min upon 3-min irradiation) than drug release from TK-NPs carrying only PTX in the same conditions (18.8%). Accelerated drug release was also observed in vitro upon irradiation, compared to non-irradiated cells. ROS produced under SDT not only killed the tumor cells directly but also indirectly by fastening the release of PTX. The synergistic effect results in a high rate of cell viability loss (89.7%) and apoptosis (68.8%) in human glioma (U87) cells and maximum tumor growth inhibition in vivo (84.5%) [86]. 

### 4.3. TK and Enzymes 

Enzymes were also exploited for dual stimulus nanosystems based on TK. For instance, Chen and co-workers developed amine-functionalized zinc oxide NPs with dual-surface modification including the cell-penetrating peptide (R8) and DOX which was attached via TK.). Positively charged NPs were then masked by hyaluronic acid (HA) for long circulation in blood as well as hyaluronidase sensitivity. Once the nanoparticles reached the tumor, the presence of high hyaluronidase (HAse) concentrations in the tumor microenvironment led to the digestion of the HA coating, exposing the cell penetrating peptides and promoting cell uptake specifically into cancer cells. In cancer cells, Zn^2+^ produced by the degradation of the ZnO NPs, which occurs only at acidic pHs (i.e., lysosomes) induced the production of ROS triggering the release of DOX in situ. Release studies in PBS buffer showed that, in presence of HAse in the release medium, DOX release at 5 h was faster (51.3%) than in absence of HAse (42% in 5 h) due to the rapid degradation of the HA shell. ZnO NPs ensured a higher tumor apoptosis rate (71.2 %) respective of free DOX (12.9%), and superior anticancer activity [87]. Another example was provided by Yin and co-workers, who prepared co-block polymers from PEG and polymerized methacrylate monomers with CPT, in which CPT has been conjugated to the side chains of methacrylate through a TK linker. These co-block polymers self-assembled into micelles encapsulating β-lapachone (Lapa), named Lapa@NPs, that produces ROS by means of NADPH quinone oxidoreductase 1 (NQO1) overexpressed in certain tumors. Lapa was exploited to increase the ROS level at the tumor site. Drug release studies in PBS buffer at different H_2_O_2_ concentrations showed that the CPT released was negligible or very slow in the absence of ROS or at low ROS concentrations (0.1 mM H_2_O_2_). This was useful to avoid unwanted drug release in normal tissues. In contrast, the release of free CPT from the NPs was significantly accelerated at higher ROS concentrations (1 mM H_2_O_2_) due to TK cleavage. CPT release studies inside cells of mouse mammary tumor cell line (4T1) revealed that a 10-fold increase of released CPT after 24 h in cells treated with Lapa@NPs compared to cells treated with CPT-NPs. Also, higher toxicity was observed for Lapa@NPs compared to free CPT in vitro (4T1 cells) and in vivo [51].

### 4.4. pH

pH is another stimulus used in association with elevated ROS levels in the diseased site. In physiological conditions, the pH is maintained around 7.4, but in certain disease conditions, such as cancer and inflammation, the microenvironment’s pH becomes acidic [88,89]. On the other hand, endosomal/lysosomal cellular compartments, as well as the stomach, are acidic. Thus, pH-responsive DDS can be exploited for the delivery of drugs in cancer, inflammation, into endo/lysosomes, as well as for the oral administration of drugs in the stomach or intestine [90].

The use of ROS and pH dual stimuli was reported for the preparation of crosslinked micelles. For instance, Zhang et al. used a co-polymer of polyethylene glycol (PEG) with two poly(aminoacids), named poly(N6-carbobenzyloxy-lysine) and poly(β-benzyl-L-aspartate) (PEG−pLys−pBla). DOX was covalently attached to the side chains of pBla by means of a pH sensitive hydrazone linker. DOX co-polymer prodrugs self-assembled into micelles which were then stabilized by crosslinking the external blocks of pLys via ROS-cleavable TK linkers. TK-based cross-linking ensured nanoparticle stability in blood circulation due to its stability at pH 7.4 and in the absence of ROS, preventing premature drug release. The hydrazine bond hydrolyzed in the tumor site at pH 5.0 freeing DOX. Finally, the high H_2_O_2_ levels in cancer cells selectively cleaved the TK moieties releasing of free DOX [91]. With a similar approach, Wang and co-workers prepared dual responsive nanomicelles made of an amphiphilic triblock co-polymer and a prodrug of PEG conjugated to doxorubicin via a TK linker (PEG-PTK-DOX). The nanomicelles were loaded with β-lapachone (Lapa) and ferric ions. The nano system transitioned from hydrophobic-to-hydrophilic in response to the pH with the release of Lapa, killing cells by a combination of toxic ROS and DOX released from the polyprodrug induced by TK cleavage [92].

### 4.5. Multistimuli 

Multistimuli-responsive nano-systems were also developed to improve the efficacy of TK-based DDS. For instance, ROS sensitive molecules were used in combination with light and pH by Bao et al. They fabricated ROS-responsive self-assembled micelles by conjugating DOX to dextran as a hydrophilic backbone through TK linker. Dextran was also modified with histidine to encapsulate the photosensitizer porphyrin (Zn-TPP) via an acid-responsive coordinated interaction. In the tumor site, the acid microenvironment (pH < 6) induced the dissociation of the acid-sensitive micelles and release Zn-TPP. ROS produced by the photosensitizer under light irradiation resulted in the cleavage of the TK linker and triggered the release of DOX. In vivo ROS-triggered DOX release studies showed significantly improved cell growth inhibitory effects with light irritation due to the combined application of photodynamic chemotherapy [93]. 

ROS-responsive DDS have also been combined with light and temperature stimuli. In particular, the localized generation of heat in the diseased tissues after exposure to light is exploited in photothermal therapy (PTT). This non-invasive technique is based on selectively accumulating a photothermal agent (PTA) at the tumor site which can convert absorbed light energy to heat when irradiated. This process induces localized heat that leads to irreversible cell damage and consequent cell death at the tumor site [94,95]. Therefore, the selective accumulation of PTAs, enable selective thermo-induced toxicity in cancer cells with minimized damage to the surrounding healthy tissues [96]. In order to benefit from the combination of light and heat stimuli, Li and co-workers developed NPs self-assembled polyethylene glycol conjugated to boron dipyrromethene (BODIPY) via a TK linker (PEG-TK-BODIPY). These NPs encapsulated iodine for the simultaneous production of ROS and heat upon irradiation [97]. In another work by Li et al., with the aim to achieve PDT treatment against hypoxic tumors, cypate and Ce6 were simultaneously conjugated to the surface of polyamidoamine (PAMAM) dendrimers to obtain CC-PAMAM DDS that respond to ROS, PDT, and temperature stimuli. In the first response, a temperature stimulus decomposed H_2_O_2_ to O_2_ alleviating the cellular hypoxia. This was followed by the second stimulus, light, to produce ROS. After these stimuli, CC-PAMAM dendrimers were released from the NPs, with deep penetration in hypoxic pancreatic tumors and PDT damage [71]. 

## 5. Current Limitations in the Application of TK-Based DDS

The research articles recently published in the literature highlight the potential of TK-based DDS mainly in cancer therapy, but also for other pathologies including inflammation. However, there are still critical aspects to consider for these kinds of applications.

Regarding the applications to cancer, some studies have demonstrated in vivo anticancer efficacy of nano systems in response to endogenous pathological concentrations of ROS. In these cases, nanoparticles made with TK present a viable option to have selective degradation and drug delivery [52,98]; however, not all cancer cells produce sufficient intracellular concentrations of ROS, or the ROS distribution is too heterogeneous, to trigger TK cleavage often limiting the effectiveness of TK-based DDS [61,99]. Thus, ROS levels must be enhanced artificially. In this regard, dual or multi-responsive TK-based DDS which combine ROS responsiveness with other environmental stimuli have been developed. Among all investigated stimuli, the use of light to produce singlet oxygen by means of photosensitizers has been the most used; not only to provide higher ROS concentrations but also to achieve synergistic effects with chemotherapeutics. Sonodynamic therapy has also been employed in an analogous fashion showing promising results that not only significantly reduced the tumor volume, but also eliminated it [71].

Other stimuli including pH and enzymes have been exploited to combine the technological advantages of ROS-sensitivity of TK with other benefits (e.g., selective cell uptake in cancer cells) in a single DDS; however, their complex design makes it difficult to perform a full physico-chemical and technological characterization of these nanosystems. This complexity may also impact the reproducibility of these formulations, which is an important factor to consider in the perspective of their clinical application. In addition, further studies to select optimal combination of loaded drug amount, photo/sonosensitizer, irradiation time, laser intensity are necessary to maximize the safety and therapeutic potential of these nano systems. Finally, although many studies have shown promising results in vitro, in vivo studies are still lacking [33,93,100].

The use of ROS-responsive material in inflammatory diseases has been less explored; however, a few research articles explored TK-based DDS for inflammatory bowel diseases with promising results. Generally, secondary stimuli have not been applied for the treatment of these diseases. This could be attributed to the fact that the concentration of ROS in inflammation conditions is much higher than in cancer. For instance, macrophages can reach intracellular concentrations ranging from 10–1000 mM [101]. Moreover, TK groups scavenge ROS. Therefore, when delivered with anti-inflammatory or/and antioxidants, a synergistic effect can be achieved. 

Regarding the safety of TK-based DDS, since one of the by-products of TK linker cleavage is the natural metabolite, acetone [102], minimal linker toxicity is expected. Furthermore, there have been no reported studies suggesting safety issues of developed TK-based DDS; however, while the therapeutic potential of such formulations has been highly documented, the biosafety of these complex nanosystems still requires additional in vivo research to guarantee clinical translation. On the other hand, the biocompatibility of ROS-responsive DDS also depends on the composition of the material, size, concentration, etc. Thus, safety should be evaluated for any designed TK-based DDS.

## 6. Conclusions and Perspectives 

The use of TK functional groups in the design of ROS-responsive DDS has grown exponentially in recent years. This is because it can be selectively cleaved upon its exposure to different types of ROS while also being considered biocompatible, biodegradable, non-toxic, and well-characterized in terms of chemistry and biological reactivity. 

In the design of these TK-containing nanosystems, different technological uses of TK have been applied to overcome the major limitations of conventional DDS counterparts. For instance, TK has been used as a linker to attach drugs to the surface of NPs or to form prodrugs self-assembled into NPs that stably hold therapeutics during delivery but release them on-demand upon ROS-specific cleavage. Alternatively, TK has been integrated into polymer chains of NPs to control degradation and drug release kinetics to prevent premature drug leakage or to ensure drug release specifically in the diseased area. Other technological uses of TK include the design of ROS-responsive gates to prevent premature drug release or its use to change the surface characteristics of the DDS for enhanced cell-uptake and site-specific drug delivery. 

All considered, a plethora of studies suggest that TK is not only a ROS-sensitive group that impart disease-targeting properties to DDS, but also a versatile tool that can be employed in different manners in the design of smart DDS to achieve multiple technological aims; however, some challenges still need to be confronted, especially regarding the translation of these systems to clinical trials. Moreover, the therapeutic potential of these nano systems has only been investigated for a narrow range of applications. The potential of TK is often not completely harnessed due to the lack of studies of its possible uses in nanotechnology. A more conscious utilization of TK may allow researchers to more effectively exploit this ROS-responsive linker and design nanomedicines with improved disease-targeting abilities, providing novel opportunities not only in the treatment of tumors, but also for other pathologies.

## Data Availability

No new data was reported.
